# Liver fibrosis is associated with cognitive impairment in HIV-positive patients

**DOI:** 10.7448/IAS.17.4.19722

**Published:** 2014-11-02

**Authors:** Nicoletta Ciccarelli, Massimiliano Fabbiani, Pierfrancesco Grima, Silio Limiti, Iuri Fanti, Annalisa Mondi, Roberta Gagliardini, Alessandro D'Avino, Alberto Borghetti, Roberto Cauda, Simona Di Giambenedetto

**Affiliations:** 1 Institute of Clinical Infectious Diseases, Catholic University of Rome, Rome, Italy; 2 Institute of Infectious Diseases, S. Caterina Novella Hospital, Galatina, Italy

## Abstract

**Introduction:**

The aim of our study was to investigate the potential relationship between liver fibrosis (LF) and cognitive performance in HIV+ patients.

**Materials and Methods:**

We performed a cross-sectional cohort study by consecutively enrolling HIV+ patients during routine outpatient visits at two clinical centres in Italy. Subjects with decompensated liver disease were excluded. All subjects underwent a comprehensive neuropsychological battery exploring memory, attention, psychomotor speed and language; cognitive impairment was defined as at least two abnormal [1.5 SD below the mean for appropriate norms] cognitive domains. LF was explored by calculating FIB4 index; in a subgroup of patients, LF was also assessed by transient elastography. Factors associated with cognitive impairment were investigated by logistic regression models.

**Results:**

A total of 413 patients [77% males, median age 46 (IQR 39–52), 17% with past AIDS-defining events, 19% past IDU, 3% with diabetes, 94% on cART, 90% with HIV RNA <50 copies/mL, 18% co-infected with HCV] were enrolled. Seventeen patients (4%) had FIB4 >3.25 and 14/129 (3%) had liver stiffness >14KPa. Forty-seven patients (11%) were diagnosed with cognitive impairment. At multivariate analyses patients with FIB4 >1.45 showed a higher risk of cognitive impairment in comparison with those with lower values (OR 2.19, 95% CI 1.02–4.72; p=0.044) after adjusting for education (OR 0.79, 95% CI 0.71–0.88; p<0.001), past IDU (OR 1.69, 95% CI 0.67–4.23; p=0.264), diabetes (OR 2.35, 95% CI 0.62–8.86; p=0.207), HIV RNA <50 copies/mL (OR 0.47, 95% CI 0.19–1.14; p=0.095) and HCV co-infection (OR 0.88, 95% CI 0.33–2.39; p=0.807). Analyzing any single cognitive domain, a higher risk of abnormal psychomotor speed was associated with fibroscan score >14KPa in comparison with fibroscan score <7KPa (OR 285.07; 95% CI 2.42–33574.06; p=0.020) after adjusting for education (OR 0.54, 95% CI 0.31–0.92; p=0.024), age (for 10 years increase) (OR 2.03, 95% CI 0.55–7.53; p=0.288), past IDU (OR 4.43, 95% CI 0.35–7.57; p=0.526), HIV RNA <50 copies/mL (OR 0.01, 95% CI 0.00–0.18; p=0.003), HIV history (for 1 year increase) (OR 0.96, 95% CI 0.83–1.12; p=0.641), CD4 cells count at nadir (OR 1.10, 95% CI 0.56–2.16; p=0.779), and HCV co-infection (OR 0.06; 95% CI 0.00–1.93; p=0.113).

**Conclusions:**

In HIV-infected patients higher LF, estimated through non-invasive methods, is associated to a higher risk of cognitive impairment.

